# Protein interaction and functional data indicate MTHFD2 involvement in RNA processing and translation

**DOI:** 10.1186/s40170-018-0185-4

**Published:** 2018-09-27

**Authors:** Costas Koufaris, Roland Nilsson

**Affiliations:** 1 Cardiovascular Medicine Unit, Department of Medicine, Karolinska Institutet, SE-171 76 Stockholm, Sweden; 2 Division of Cardiovascular Medicine, Karolinska University Hospital, SE-171 76 Stockholm, Sweden; 3 Center for Molecular Medicine, Karolinska Institutet, SE-171 76 Stockholm, Sweden

**Keywords:** Interactome, Moonlighting, Heat shock proteins, RNA, Non-metabolic

## Abstract

**Background:**

The folate-coupled metabolic enzyme MTHFD2 is overexpressed in many tumor types and required for cancer cell proliferation, and is therefore of interest as a potential cancer therapeutic target. However, recent evidence suggests that MTHFD2 has a non-enzymatic function which may underlie the dependence of cancer cells on this protein. Understanding this non-enzymatic function is important for optimal targeting of MTHFD2 in cancer.

**Methods:**

To identify potential non-enzymatic functions of MTHFD2, we defined its interacting proteins using co-immunoprecipitation and mass spectrometry and integrated this information with large-scale co-expression analysis, protein dynamics, and gene expression response to MTHFD2 knockdown.

**Results:**

We found that MTHFD2 physically interacts with a set of nuclear proteins involved in RNA metabolism and translation, including components of the small ribosomal subunit and multiple members of the RNA-processing hnRNP family. Interacting proteins were also in general co-expressed with MTHFD2 in experiments that stimulate or repress proliferation, suggesting a close functional relationship. Also, unlike other folate one-carbon enzymes, the MTHFD2 protein has a short half-life and responds rapidly to serum. Finally, shRNA against MTHFD2 depletes several of its interactors and yields an overall transcriptional response similar to targeted inhibition of certain ribosomal subunits.

**Conclusions:**

Taken together, our findings suggest a novel function of MTHFD2 in RNA metabolism and translation.

**Electronic supplementary material:**

The online version of this article (10.1186/s40170-018-0185-4) contains supplementary material, which is available to authorized users.

## Background

**Fig. 1:**
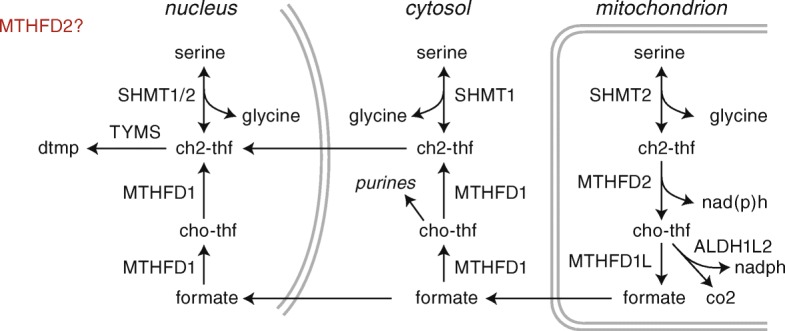
Schematic diagram of enzymes, metabolites, and compartmentalization of folate one-carbon metabolism. One-carbon metabolism enzymes are now known to be present and active in three distinct compartments, the nucleus, mitochondria, and cytosol, linked by the flow of formate between them. The function of MTHFD2 in the mitochondria is well understood, although its function in the nucleus is not known

Altered metabolism is a hallmark of cancer cells that facilitates their growth and survival [1]. In particular, metabolism of folate-coupled one-carbon groups is reprogrammed in a wide variety of cancers [2–4]. One-carbon metabolism is localized into cytosolic, mitochondrial, and nuclear compartments (Fig. 1) and is required for the synthesis of purines, dTMP, and remethylation of homocysteine to methionine. Extensive evidence now supports that proliferating cancer cells primarily rely on the mitochondrial catabolism of serine via SHMT2 as their main source of one-carbon groups [5–7], rather than the corresponding cytosolic enzymes. The reason for this preference is unclear, but may be due to the mitochondrial NADH/NAD ratio favoring flux in the oxidative direction [8], or related to the associated reduction of NAD and NADP [7]. In the nucleus, the formate produced by the mitochondrial pathway supports synthesis of dTMP during DNA replication.

Within mitochondrial folate one-carbon metabolism, the enzyme MTHFD2 has attracted considerable attention as a potential target for cancer therapeutics [4, 9, 10], motivated by a favorable expression profile with high expression in various human tumor types [4] but low or undetectable levels in most adult tissues. The normal function of MTHFD2 may be in embryogenesis, since the enzyme has long been known to be highly expressed and essential during embryonic development [11], and a paralogous mitochondrial enzyme, MTHFD2L, is expressed in normal adult tissues [12]. Expression of MTHFD2 in tumors also correlates with poor disease outcome in breast cancer [13], liver cancer [14], and acute myeloid leukemia (AML) [10]. Knockdown of MTHFD2 can inhibit cancer cell proliferation in vitro [4, 10, 15] and reduces (but does not abolish) tumor growth in vivo [7, 10]. This combination of specific expression in transformed cells and severe cell phenotypes upon knockdown render MTHFD2 a compelling potential drug target.

Nevertheless, several observations suggest that the known metabolic function of MTHFD2 may not be the sole explanation for its prominent role in cancer. First, supplementing cultures with formate, the primary product of the mitochondrial pathway (Fig. 1), fails to rescue MTHFD2 knockdown cancer cells [4, 10], indicating that another function of the MTHFD2 protein might be necessary for growth. Second, cells can rapidly adapt to loss of MTHFD2 by shifting to the cytosolic one-carbon pathway [7], suggesting a degree of redundancy where flux through the mitochondrial pathway is not strictly essential. Third, knockdown of the SHMT2 enzyme, which should also block the mitochondrial pathway, does not cause cell death unless glycine is removed [2, 16]. Moreover, the MTHFD2 protein was recently found to be present in the nucleus at sites of newly synthesized DNA, and over-expression of catalytically inactive MTHFD2 drives cell proliferation [17], suggestive of a role in signaling/regulation rather than metabolism. Knockdown of MTHFD2 also affects invasion and migration in vitro [14, 18], although no obvious mechanistic link exists between one-carbon metabolism and these phenotypes.

Given these observations, it is becoming clear that the effective targeting of the MTHFD2 protein requires a better understanding of its role in cancer cells beyond metabolism. Although chemical inhibitors of the MTHFD enzymatic activity have been reported [19, 20], different strategies may be required if a non-enzymatic function of the MTHFD2 enzyme is critical for cancer cells. For these reasons, we performed an initial investigation of the possible non-metabolic functions of MTHFD2 by mapping the protein’s interacting partners, co-expression pattern and the transcriptional responses to knockdown. Taken together, our results suggest a previously unrecognized role for MTHFD2 in RNA metabolism and translation.

## Methods

### Cell culture

HCT-116 cell lines were obtained from the National Cancer Institute. The MTHFD2 CRISPR^−/−^ knockout (D2-KO) and parental wildtype (WT) HCT-116 cells were obtained from the lab of Dr. Rabinowitz [7]. Cells were grown in RPMI-1640 medium supplemented with 5% FBS (Life technologies) and Penicillin/Streptomycin (Gibco) and maintained at 37 °C and 5% CO2.

For serum starvation experiments, 200,000 cells were plated in six-well plates. The next day, the medium was removed, the cells were washed with PBS and then serum-free RPMI-1640 medium was added. Cyclohexamide was obtained from Sigma Aldrich (C4859). For the serum re-stimulation experiments, after 48 h of serum starvation, wells were washed with PBS and then fresh RPMI-1640 containing 20% FBS was added to the cells.

### Real-time PCR

RNA was isolated from cells using the Qiagen RNeasy mini kit and quantified using a Nanodrop ND-1000 spectrophotometer. The RNA was reverse transcribed to cDNA using the VILO cDNA synthesis kit (Invitrogen). The qPCR reactions were performed in triplicate on a StepOne Real-Time PCR machine (Thermo Fisher Scientific) using the Fast SYBR Green master mix (Thermo Fisher Scientific). Oligos were ordered from Sigma Aldrich. Primer sequences are listed in Additional file 1. Relative amounts of mRNA were calculated using the ΔΔC_T_ method normalized to RPLPO mRNA as a reference.

### Immunoblotting

Total protein was isolated from cells using RIPA buffer (Thermo Fisher Scientific), with the addition of × 100 Halt protease inhibitors (Thermo Fisher Scientific) just prior to the extraction. Protein from nuclear and cytosolic cellular compartments were fragmented as described previously [17]. Protein levels were quantified by the BCA assay (Pierce). Equal amounts of protein (10–20 μg) were loaded per lane of 10% SDS-PAGE gel. The following primary antibodies were incubated overnight: anti-MTHFD2 (Proteintech 12270-1-AP); anti-MTHFD1 (Proteintech 6113-1-AP); anti-MTHFD1L (Proteintech 10794-1-AP); Lamin A/C (Thermo Fisher Scientific MA3-1000); HSP60 (Abcam ab46798); Tubulin (Abcam ab6046); and COX IV (Abcam 33985). Quantification of protein bands was performed using the ImageJ software and values were normalized to tubulin for each sample.

### Co-immunoprecipitation

WT and D2-KO HCT-116 cells were lysed using the moderate Pierce IP lysis buffer (Thermo Fisher Scientific) with addition of × 100 Halt protease inhibitors (Thermo Fisher Scientific). All steps were performed at 4 °C to reduce protein disassociation. The protein lysate was quantified using the BCA assay, and 1 or 2 mg of lysate was used for immunoprecipitation with no cross-linkage. Immunoprecipitation was performed using two separate anti-MTHFD2 antibodies (Proteintech 12270-1-AP (AP) and Genetex N1C3 (NC)) or an anti-rabbit immunoglobulin (IgG) (Santa Cruz). The immunoprecipitation was performed at 4 °C for 2 h using Dynabeads Protein A (Thermo Fisher Scientific) following the manufacturer’s instructions.

### In-gel digestion and mass spectrometry

Immunoprecipitated proteins were eluted from Dynabeads through a combination of elution buffer and heating and separated on a 10% SDS gel. In-gel staining of proteins was performed using the SilverQuest silver staining kit (Thermo Fisher Scientific). Protein lanes were excised manually and in-gel digested using a MassPREP robotic protein-handling system (Waters, Millford, MA, USA). Gel pieces were distained twice with 100 μL of 50 mM ammonium bicarbonate (AmBic) containing 50% acetonitrile at 40 °C for 10 min. Proteins were reduced with 10 mM DTT in 100 mM Ambic for 30 min at 40 °C and alkylated with 55 mM iodoacetamide in 100 mM AmBic at 40 °C for 20 min followed with in-gel digestion with 0.3 μg trypsin (sequence grade, Promega, Madison, WI, USA) in 50 mM at 40 °C AmBic for 5 h. The tryptic peptides were extracted with 1% formic acid in 2% acetonitrile, followed by 50% acetonitrile twice. The liquid was evaporated to dryness, and the peptides were reconstituted in 10 μL of 2% acetonitrile/0.1% formic acid. Five microliters of the samples was injected onto the LC-MS/MS system (Ultimate™ 3000 RSLCnano chromatography system and Q Exactive HF Orbitrap mass spectrometer, Thermo Scientific). The peptides were separated on an Easy-C18 column, 50 cm (Thermo Fisher Scientific) at 55 °C in a 120-min gradient at a flow rate of 300 nL/min. The linear gradient was from 5 to 26% of buffer B (98% acetonitrile/0.1% formic acid) in 115 min and to 95% of buffer B in 5 min.

We used data-dependent acquisition with a survey scan range of 300 to 1650 m/z, at a resolution of 120,000 m/z and selected up to 16 most abundant features with a charge state ≥2 for HCD fragmentation at a normalized collision energy of 26 and a resolution of 30,000 at m/z 200. To limit repeated sequencing, dynamic exclusion of sequenced peptides was set to 90 s. Thresholds for ion injection time and ion target values were set to 250 ms and 5 × 106 for the survey scans, and 120 ms and 2 × 105 for the MS/MS scans. Data were acquired using the Xcalibur software (Thermo Fisher Scientific). The spectra were analyzed using the Mascot search engine v. 2.4 (Matrix Science Ltd., UK) using carbamidomethylation (C) as fixed and deamidation (NQ), oxidation (M) as variable modification. The precursor mass tolerance was set to 10 ppm while MS/MS tolerance was 0.02 Da allowing 2 missed cleavages. The SwissProt database was used with taxonomy limited to *Homo sapiens*.

In total, we performed 12 Co-IP experiments (for HCT-116 WT, 4 using AP antibody, 2 using NC antibody, and 2 using IgG antibody; for HCT-116 D2-KO, 2 using AP antibody and 2 using NC antibody) followed by MS analysis. A protein was considered as high confidence if at least two unique peptides were detected in 50% of IP-MS samples for each of the MTHFD2 antibodies used and was not detected in any of the IP-MS samples from D2-KO cells, nor the IgG isotype control IP-MS samples.

### Protein-protein interaction network

A protein-protein interaction network was extracted from the STRING database v10.5 [21] using the set of MTHFD2 interacting proteins identified here. Only experimental data (e.g., from co-purifications and yeast two hybrids) imported in STRING from primary sources were used for constructing the network. Threshold for connections between proteins was set at medium score (more than 0.400) as calculated in STRING, and gene ontology (GO) enrichment was calculated.

### Half-life analysis

For half-life analysis, we used publicly available data on global protein half-life in NIH3T3 cells [22], HeLa, and C2C12 cells [23]. The list of 1652 proteins involved in metabolism were obtained from Recon2 [24] model of human metabolism, of which 596 were measured in HeLa, 575 in C2C12, and 605 in NIH3T3.

### Co-expression analysis

Calculation of gene co-expression was performed using a clustering-based method, as described previously [17]. Briefly, we analyzed 25,485 genes represented on a variety of microarray platforms for co-expression with MTHFD2 across 8097 human, mouse and rat data sets obtained from the Gene Expression Omnibus (GEO). From the resulting matrix of coexpression scores *x*_*gd*_, we computed the overall coexpression score *w*_*g*_ for each gene *g* by simply summing over all data sets *d*. We selected the 50 data sets showing highest coexpression for the D2PPI genes by scoring each data set by the weighted sum $\sum \limits_g{w}_g{x}_{gd}$, where *g* ranges over the 29 genes in the D2PPI gene set; and similarly for the ATF4 gene set (Fig. 3b). Example data sets from GEO (Fig. 3c–f) were analyzed as deposited on GEO, with no further normalization. In cases where multiple probes against a single gene were present on the arrays, the probe with highest mean signal across all samples was used. Enrichment analysis was done using the GSEA-P statistic [25], and an enrichment *p* value was calculated by gene permutation (10,000 permutations). An independent analysis was made using the SEEK tool (http://seek.princeton.edu) with default settings [26].

### Connectivity Map (CMap) analysis

Expression data from the Connectivity Map (CMap) project in Level 5 (signature) GCTX format was obtained from GEO (accession GSE92742), and data for 99 experiments with five distinct shRNA hairpins against MTHFD2 across 12,328 measured genes was extracted using R v.3.3.3 and the cmapR package (https://github.com/cmap/cmapR). Since the three hairpins TRCN0000036550, TRCN0000036551, and TRCN0000036553 exhibited strongest knockdown on MTHFD2 itself, these were used for subsequent analysis. Data from the ASC and NPC cell lines were discarded due to few replicates. The z-scores for the remaining 9 cell lines were averaged into a final *z*-score vector, which was used for enrichment analysis using the GSEA-P statistic [25]. The genesets examined were downloaded from ConsensusPathDB collection (http://cpdb.molgen.mpg.de/). An enrichment *p* value was calculated by gene permutation (10,000 permutations).

Connectivity scores between hairpins targeting three one-carbon enzymes included in CMap (MTHFD2, MTHFD1, and SHMT1) and hairpins targeting 3799 examined genes were analyzed using the CMap online tools (https://clue.io/cmap). Connectivity scores of more than 90 or less than −90 were considered as significantly enriched. Clustering analysis of connectivity scores was performed using ClustVis [27] with average linkage and correlation metric. Ribosomal genes were selected for analysis based on the KEGG ribosome gene set (entry hsa03010).

### RNAi and CRISPR screening data analysis

Global RNAi and CRISPR screening data were performed as part of the Achilles project in 501 and 342 cell lines respectively [28, 29]. We also examined a second CRISPR study conducted in 14 AML cell lines [30]. To identify genes with similar dependencies across examined cell lines in the RNAi and CRISPR screens, we calculated Pearson’s correlation for MTHFD2 against all examined genes in both datasets.

## Results

### Protein interaction partners of MTHFD2

**Fig. 2:**
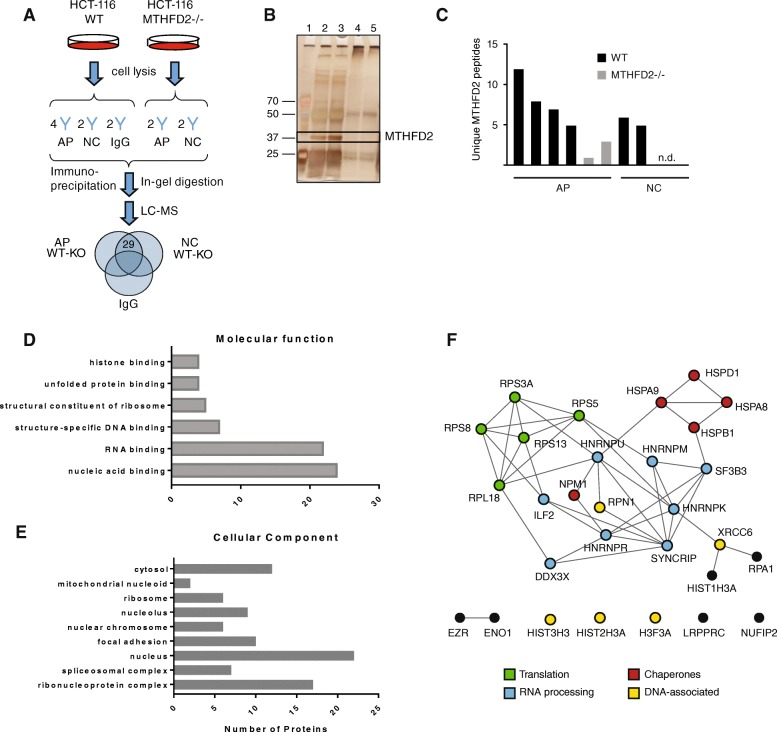
Defining the MTHFD2 interactome. **a** Schematic of the strategy used for identifying MTHFD2 interactors. Immunoprecipitation was performed on parental HCT-116 cells (WT) and MTHFD2 knockout cells (D2-KO) using either one of two anti-MTHFD2 antibodies (AP and NC) or an anti-rabbit immunoglobulin (IgG) antibody. The numbers above antibody names indicate the number of replicate experiments performed for a given condition. After in-gel digestion and MS 29 proteins were found to be detected in IP from both anti-MTHFD2 antibodies in parental HCT-116 cells but not in IP performed in MTHFD2 knockout cells or using IgG antibody. **b** Representative silver-stained SDS-gel containing co-immunoprecipitated samples. Lane 1 contained a molecular weight ladder. Lane 2 and 3 lysates from wildtype HCT-116 cells after immunoprecipitation with anti-MTHFD2 antibody, lanes 4 and 5 lysates from same cells immunoprecipitated using anti-rabbit IgG antibody. **c** Number of unique peptides matching to MTHFD2 identified in replicate experiments with two anti-MTHFD2 antibodies in WT or knockout D2-KO cells. **d**, **e** Selected significantly enriched GO Molecular Function and Cellular Component respectively. **f** PPI network generated for 29 identified MTHFD2 interactors using experimental evidence in STRING database and medium confidence links (> 0.400). Nodes with no interaction are not shown. Thickness of edges represent strength of evidence supporting interaction. Members of protein functional groups are indicated according to color chart

Since a non-metabolic function of MTHFD2 would likely involve direct physical contact with other proteins, we decided to first perform co-immunoprecipitation (Co-IP) of MTHFD2-interacting proteins followed by mass spectrometry (MS) to identify its binding partners. To avoid potential artifacts due to unspecific antibody binding, we chose to perform Co-IP experiments in wildtype (WT) and CRISPR MTHFD2 knockout (D2-KO) HCT-116 cells [7], using two distinct MTHFD2 antibodies, 12270-1AP (AP; Proteintech) and N3C3 (NC; Genetex) as well as an isotype control (IgG) antibody (Fig. 2a**)**. We first confirmed by immunoblotting and IP that the MTHFD2 protein was absent from D2-KO cells (Additional file 2). Next, we performed Co-IP followed by SDS-PAGE and silver-staining to verify the efficient immunoprecipitation of MTHFD2. This analysis revealed a strong band at the expected size of MTHFD2, as well as a large number of other immunoprecipitated protein bands (Fig. 2b). In D2-KO cells, the MTHFD2 band was not detected, but other protein bands were still observable, presumably due to non-specific binding of the antibodies used (Additional file 2). Detected peptide fragments in all examined Co-IP lysates are listed in Additional file 3. Reassuringly, MTHFD2 itself was reliably identified from WT cells using both antibodies and strongly decreased in the D2-KO lysates (Fig. 2c).

We defined high-confidence MTHFD2-interacting proteins as those that were reliably detected by Co-IP in WT but not D2-KO cells, using both the AP and NC antibodies, and were also absent from IgG lysates (Fig. 2a; Methods). With these criteria, we identified 29 MTHFD2-interacting proteins, from here on referred to as the D2PPI gene set (Table 1). The majority of these proteins have been reported to localize to the nucleus, consistent with previous evidence that MTHFD2 is also a nuclear protein [17]. While two mitochondrial RNA-binding proteins were present (LRPPRC; HSPA9), we found no other one-carbon enzymes among the D2PPI proteins. We did detect MTHFD1 using the AP antibody, but this protein was found in D2-KO cells as well, indicating that this interaction is due to unspecific binding. Hence, MTHFD2 does not appear to participate in the previously reported nuclear thymidylate synthesis complex [31].

**Table 1:** MTHFD2 protein interactors identified by CoIP and MS

Symbol	Name	AP	NC	RNA binding	Unfolded protein binding	DNA Binding
HSPA8	Heat shock cognate 71 kDa protein	3/4	1/2	X	X	
HSPA9	Stress-70 protein, mitochondrial	3/4	1/2	X	X	
HSPB1	Heat shock protein beta-1	3/4	1/2	X		
HSPD1	60 kDa heat shock protein	3/4	1/2	X	X	X
NPM1	Nucleophosmin	2/4	1/2	X	X	
HIST1H3A	Histone H3.1	2/4	1/2			X
HIST2H3A	Histone H3.2	2/4	1/2			X
HIST3H3	Histone H3.1 t	2/4	1/2			X
H3F3A	Histone H3.3	2/4	1/2			X
XRCC6	X-ray repair cross-complementing protein 6	2/4	1/2	X		X
RPA1	Replication protein A 70 kDa DNA-binding subunit	2/4	1/2			X
HNRNPK	Heterogeneous nuclear ribonucleoprotein K	2/4	1/2	X		X
HNRNPM	Heterogeneous nuclear ribonucleoprotein M	3/4	1/2	X		
SYNCRIP	Heterogeneous nuclear ribonucleoprotein Q	2/4	1/2	X		
HNRNPR	Heterogeneous nuclear ribonucleoprotein R	2/4	1/2	X		
HNRNPU	Heterogeneous nuclear ribonucleoprotein U	2/4	2/2	X		X
SF3B3	Splicing factor 3B subunit 3	2/4	1/2	X		
LRPPRC	Leucine-rich PPR motif-containing protein, mitochondrial	3/4	1/2	X		X
ILF2	Interleukin enhancer-binding factor 2	2/4	1/2	X		X
NUFIP2	Nuclear fragile X mental retardation-interacting protein 2	2/4	1/2	X		
DDX3X	ATP-dependent RNA helicase DDX3X	2/4	1/2	X		X
RPL18	60S ribosomal protein L18a	2/4	1/2	X		
RPS13	40S ribosomal protein S13	2/4	1/2	X		
RPS3A	40S ribosomal protein S3a	2/4	1/2	X		
RPS5	40S ribosomal protein S5	2/4	1/2	X		
RPS8	40S ribosomal protein S8	2/4	1/2	X		
ENO1	Alpha-enolase	2/4	1/2	X		X
EZR	Ezrin	2/4	1/2	X		
RPN1	Dolichyl-diphosphooligosaccharide--protein glycosyltransferase subunit 1	2/4	1/2	X		

Numbers indicate the unique peptides mapped to protein in given CoIP replicate

Gene Ontology (GO) analysis of these proteins confirmed a highly significant enrichment of RNA binding proteins (24/29 proteins; *q* < 10^−20^) (Fig. 2d; Additional file 4) and that a large fraction of these proteins were also nuclear (Fig. 2e). To determine if the D2PPI proteins belonged to any known complexes, we extracted a protein-protein interaction (PPI) network over these 29 proteins from the STRING database [21]. We obtained 47 physical interactions, which is two times more than expected by chance (*p* < 10^−7^); hence, the D2PPI proteins are likely part of previously known protein complexes, including the small ribosomal subunit proteins (four RPS proteins), RNA processing proteins (several hnRNP family members; SF3B3; ILF2), protein chaperones (several heat shock proteins; NPM1), and histone/DNA repair proteins (three histone proteins; XRCC6; RPN1) (Fig. 2f). Hence, the proteins shown here to directly interact with MTHFD2 indicate a role for the protein in metabolism and translation of RNA.

### Co-expression analysis of MTHFD2 and interacting proteins

**Fig. 3:**
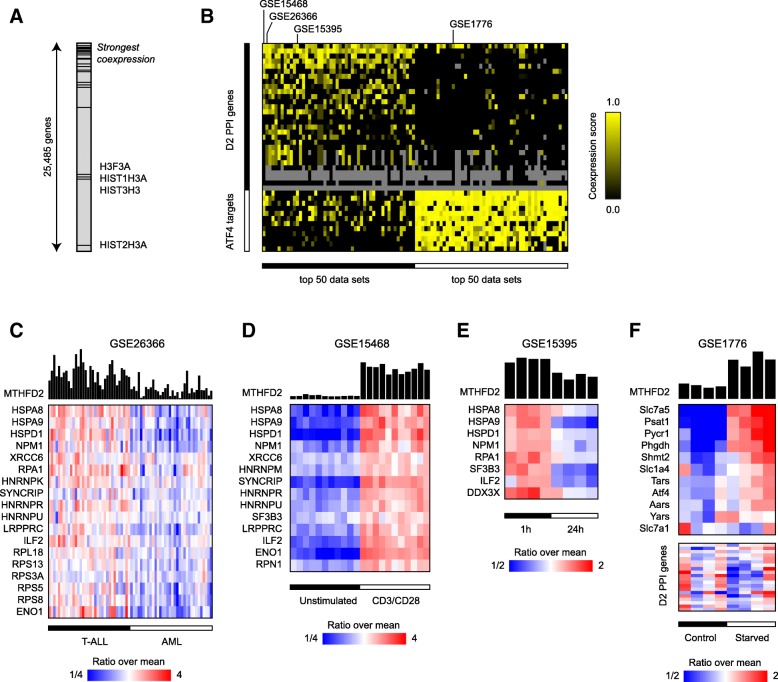
MTHFD2-interacting proteins are coexpressed with MTHFD2 in response to stimuli. **a** Enrichment plot showing the ranks of MTHFD2-interacting (D2PPI) proteins ordered by overall coexpression with MTHFD2 across 8067 gene expression data sets (see Methods). **b** Heat map of coexpression score for the D2PPI and ATF4 gene sets, across the 50 data sets exhibiting with strongest coexpression for each set. Data sets selected for panels **c**–**f** are indicated by arrows. **c**–**f** Heat maps of expression level for selected data sets. For each gene, ratio over the overall mean is shown, according to scale bars. Only genes with nonzero coexpression scores are shown. MTHFD2 expression level is shown above in linear scale, with baseline equal to zero (arbitrary units)

To systematically assess if the D2PPI proteins indeed share a common function with MTHFD2, we next asked whether their mRNAs are also frequently co-expressed with MTHFD2. We have previously shown that frequent co-expression across diverse experimental conditions is highly predictive of closely related biological function [32, 33]. Using a previously described method [17], we scored 25,845 genes for co-expression with MTHFD2 in 8067 human, rat, and mouse microarray datasets (Additional file 5). Remarkably, D2PPI gene set was clearly enriched for coexpression with MTHFD2 (*p* = 0.003, permutation test), with 13 of 29 of genes within the 95th percentile of coexpression scores (Fig. 3a). An exception was the histone proteins H3F3A, HIST1H3A, HIST3H3, and HIST2H3A that did not exhibit co-expression with MTHFD2; however, these proteins were not well-represented on the microarrays used, so this result may reflect low power to detect coexpression. An independent analysis of 3356 human gene expression datasets using the SEEK gene co-expression analysis tool [26] gave similar results (Additional file 5**)**. To investigate factors that might drive the observed coexpression of the D2PPI gene set, we identified specific gene expression data sets where this set was coexpressed (Fig. 3b, Additional file 6). Among the top scoring data sets were several tumor gene expression studies, where expression varied between subtypes; for example, in one leukemia study, highest expression was found in T-ALL and lowest in AML subtypes, suggesting specific disease contexts where the D2PPI proteins may be relevant (Fig. 3c). We also noted a concerted induction of the D2PPI proteins in stimulated T cells (Fig. 3d), in line with previous observations of MTHFD2 induction in this setting [4], and also suppression of D2PPI genes in HCT-116 cancer cells in response to treatment with a CDK inhibitor (Fig. 3e). These data indicate that MTHFD2 and the D2PPI genes are frequently coexpressed and responsive to mitogenic stimuli and anti-proliferative drugs.

As previously shown [17], a set of ATF4-responsive mRNAs involved in amino acid metabolism and aminoacyl-tRNA synthesis, including the mitochondrial folate-coupled enzymes SHMT2 and MTHFD1L, was also strongly co-expressed with MTHFD2. However, we found that these genes co-express with MTHFD2 in different conditions than the MTHFD2-interacting proteins (Fig. 3b, Additional file 6), suggesting that the underlying mechanism is different. For example, the mitochondrial enzymes were induced in contexts of amino acid starvation, possibly reflecting the involvement of this pathway in amino acid synthesis, while the D2PPI genes were not (Fig. 3f). In summary, the D2PPI genes are co-regulated with MTHFD2 in specific biological conditions, providing independent evidence that they share a biological function with MTHFD2, which appears to be distinct from ATF4-driven induction of MTHFD2.

### MTHFD2 is a rapidly regulated protein with a short half-life

**Fig. 4:**
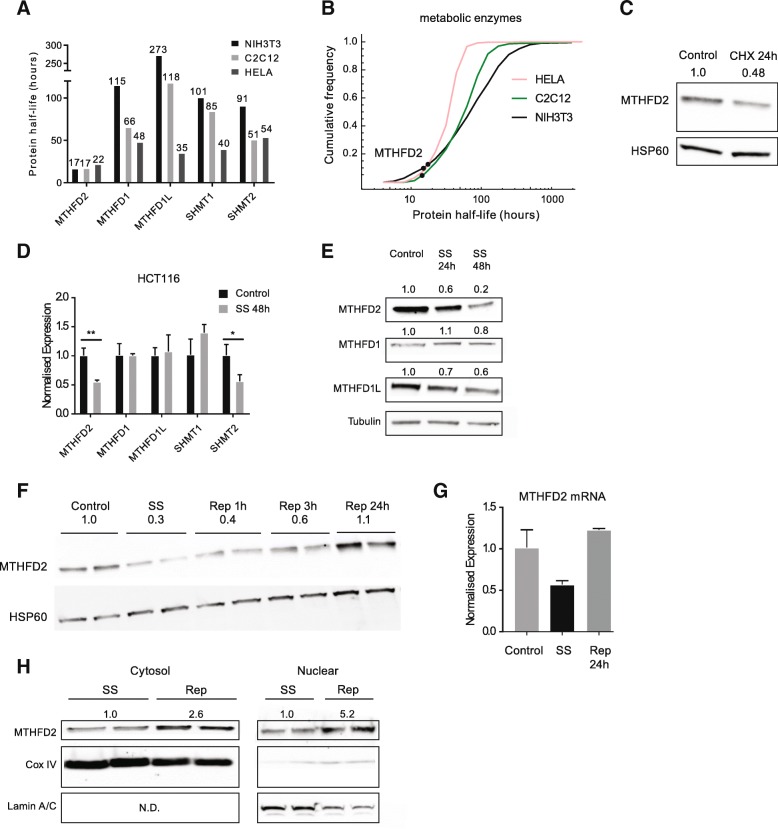
MTHFD2 is a short-lived and dynamically regulated protein. **a** Half-life of enzymes of the one-carbon pathway in in HeLa, C2C12, and NIH3T3 cell lines. **b** Cumulative frequency of all quantified enzymes (596, 576, and 605 respectively) half-life in three cell lines. Dotted lines indicate relative half-life of MTHFD2 in each cell line. **c** Effect of treatment of HCT-116 cells for 24 h with 25 mg/ml protein translation inhibitor cyclohexamide on MTHFD2 protein levels. **d** qRT-PCR measurement of effect of 48 h serum starvation on mRNA of folate one-carbon enzymes. **e** Immunoblots of MTHFD1, MTHFD1L, MTHFD2, and beta-tubulin in HCT-116 grown in normal media or serum starved for 24 or 48 h. **f** Immunoblot showing time course of MTHFD2 protein response to serum replenishment in HCT-116 cells. SS, serum-starved; rep, serum replenishment (time point indicated). **g** Quantification of MTHFD2 mRNA in HCT-116 cells in control media, serum starved (SS) for 48 h, and serum starved followed by 24 h serum replenishment (rep). **h** Levels of MTHFD2 protein in nuclear and cytosolic compartments in HCT-116 cells that were serum starved for 48 h or in replenished media for 24 h after serum starvation. The COX IV and Lamin were used as cytosolic and nuclear markers respectively. Numbers in **f** and **g** indicate fold changes relative to control

Given the above finding that MTHFD2 and the D2PPI gene set is acutely regulated by pro- and anti-proliferative stimuli, we examined in more detail the dynamics of MTHFD2 regulation. Generally, metabolic enzymes are long-lived proteins, while proteins involved in RNA metabolism, cell cycle, and signaling have shorter half-lives [22]. In a reanalysis of global protein half-life data [22, 23], we noted that the half-life of the MTHFD2 protein was consistently within 17–22 h in HeLa, C2C12, and NIH3T3 cells, which was markedly lower than the other folate one-carbon enzymes (Fig. 4a) and within the 5–10th percentile of all detected enzymes (as defined by Recon2 [24]; 596–605 enzymes were detected across three cell lines) (Fig. 4b). We confirmed this short half-life of MTHFD2 by treatment with the cyclohexamide inhibitor of protein synthesis, which resulted in an approximate halving of protein levels after 24 h (Fig. 4c). Therefore, MTHFD2 is unusually short-lived for a metabolic enzyme in general, and one-carbon enzymes in particular.

Considering the short half-life of MTHFD2, we next examined the regulation of the MTHFD2 protein compared to other enzymes in the mitochondrial folate pathway. In HCT-116 cells, after 48 h serum starvation, MTHFD2 and SHMT2 mRNA was significantly decreased, while SHMT1, MTHFD1, and MTHFD1L was unaffected (Fig. 4d). At the protein level, MTHFD2 and MTHFD1L decreased noticeably at the 24 h time point, while MTHFD1 did not (Fig. 4e), consistent with the shorter half-life of MTHFD2. In serum restimulated HCT-116 cells, MTHFD2 protein was induced within 3 h (Fig. 4f), consistent with our previous report [17]. Moreover, induction of the protein was observed in both the nuclear and cytoplasmic compartments (Fig. 4h), and also on the mRNA level (Fig. 4g). Our data show therefore that enzymes of the one-carbon pathway differ in their response to serum depletion and stimulation, with MTHFD2 being particularly responsive.

### Transcriptional response to MTHFD2 knockdown in cancer cells

**Fig. 5:**
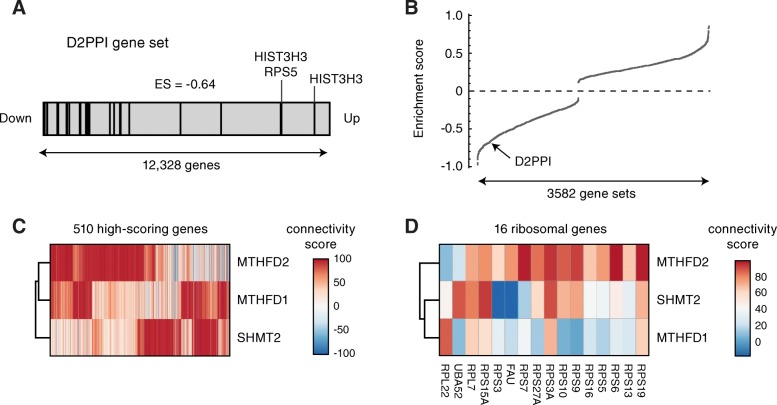
Consequences of MTHFD2 knockdown. **a** Enrichment plot showing ranks of the D2PPI gene set among 12,328 genes responding to treatment with MTHFD2 shRNA in CMap study. Overall enrichment score was − 0.64. Genes with low enrichment scores (histones and RSP5) are indicated. **b** Distribution of enrichment scores for 3582 gene sets, with arrow indicating position of D2PPI gene set. **c** Heatmap showing connectivity scores for all 510 genes with significant associations (scores > 90 or < − 90) in CMap project with either of MTHFD2, MTHFD1 or SHMT2. Genes were clustered by average linkage and Pearson correlation distance. **d** Connectivity scores between MTHFD2, MTHFD1, and SHMT2 with ribosomal genes measured in the CMap project

To study the effects of loss of MTHFD2, and how these relate to its identified interacting proteins, we analyzed transcriptomics data from MTHFD2 shRNA knockdown cells generated by the Connectivity Map (CMap) project [34]. Interestingly, the D2PPI gene set was clearly decreased (ES -0.64, *p* < 10^−4^) in MTHFD2 knockdown cells (Fig. 5a) and was among the most negatively enriched among examined collection of 3581 gene sets (Fig. 5b). Hence, loss of MTHFD2 affects expression of the D2PPI genes, consistent with a shared function.

The CMap data also allows assessing functional relationship between genes by scoring the similarity of transcriptional response of cancer cells to individual gene knockdown. In this regard, knockdown of MTHFD2 was highly similar (score > 90/100) to four out of the 14 D2PPI genes represented in CMap: HSPA8, HSPA9, HSPD1, and RPS3A. In contrast, with this criterion, other folate-metabolizing enzymes present in the CMap data set (MTHFD1, SHMT2, GLDC, AMT, TYMS, MTR, MTRR, FTCD, and DHFR) were not similar to MTHFD2, with the exception of MTHFD1 (score = 91.0, rank 414/3798), possibly reflecting the fact that loss of MTHFD1 also gives severe growth phenotypes [7]. In particular, SHMT2 was not closely related (score = 18.74 of 100, rank 2790/3798), consistent with the observations that loss of MTHFD2 is often detrimental to cells [4], while SHMT2 is not [2], and suggesting that at least some effects of MTHFD2 suppression are unrelated to mitochondrial folate metabolism.

Finally, we investigated the sets of genes that in CMap were highly similar to MTHFD2. We noted that, across all 3798 genes for which shRNA data was available in the CMap data set, MTHFD2 was highly similar (absolute score > 90) to 510 genes, compared to only 266 for SHMT2 and 169 for MTHFD1, indicating that loss MTHFD2 induces a response that more commonly occurs with gene knockdown. In addition, the set of 510 genes similar to MTHFD2 in this respect were clearly different from those of SHMT2 and MTHFD1 (Fig. 5c), again consistent with MTHFD2 having a distinct function. In particular, knockdown of MTHFD2, but not SHMT2 or MTHFD1, was highly similar to knockdown of a group of ribosomal proteins (Fig. 5d). Therefore, MTHFD2 is unique in that suppression of this protein causes cellular transcriptomic responses that are highly similar to those observed following the targeting of ribosomal proteins.

### Comparison of RNAi and CRISPR suppression of MTHFD2

**Fig. 6:**
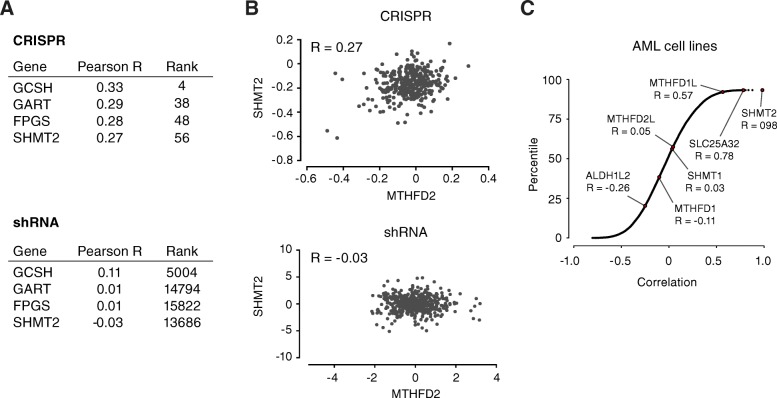
Comparison of consequences of CRISPR knockout and RNAi knockdown of MTHFD2. **a** Pearson correlation and global rank between MTHFD2 and genes involved in folate metabolism in shRNA and CRISPR screens. **b** Comparison of growth effects caused by depletion of MTHFD2 and SHMT2 using shRNA and CRISPR in Achilles project. Each circle represents a tested cell line. Pearson’s correlation is shown. **c** Distribution of correlations between growth phenotype effects of all examined genes and MTHFD2 across 14 AML cell lines. Selected genes involved in one-carbon metabolism are indicated by arrows

At first, the observation that MTHFD2 knockdown is similar to knockdown of ribosomal proteins, but not the upstream enzyme SHMT2, seemed counter-intuitive. A possible explanation is that shRNA knockdown cells still contain some residual protein, which may be sufficient to retain activity of a metabolic enzyme, but disrupts a more sensitive non-metabolic function such as control of translation. Indeed, there is evidence that a small amount of residual enzymatic activity can be sufficient to maintain necessary metabolic output, as seen for example with SHMT2 and formylmethionyl-tRNA pools [35]. To investigate this hypothesis, we compared the effects of shRNA knockdown to effects of CRISPR knockout, using genome-wide shRNA and CRISPR screening data from the Achilles project, measuring growth phenotypes across 501 and 341 cell lines, respectively. Here, genes with shared functions are expected to have similar dependency profiles across cell lines [30]. Overall, 977 genes were similar to MTHFD2 in the shRNA screening data, and 161 with CRISPR screening (Pearson’s correlation, Bonferroni-corrected *p* < 0.05). Interestingly, genes involved in folate metabolism (SHMT2, GART, GCSH, FPGS) were similar to MTHFD2 in the CRISPR, but not in the shRNA screens (Fig. 6a, b**,** Additional file 7). Moreover, in a similar analysis of an independent CRISPR dataset comprising 14 AML cell lines, SHMT2, SLC25A32, and MTHFD1L were strongly correlated with MTHFD2, while cytosolic one-carbon enzymes showed weak correlations (Fig. 6c). However, in these data sets, the D2PPI proteins frequently showed strong growth phenotypes across all cell lines and were not significantly correlated with MTHFD2 (data not shown). This is consistent with the notion that in CRISPR MTHFD2 knockouts, the metabolic disruption dominates the effects on cell proliferation, while for shRNA knockdown, other functions of MTHFD2 may play a role.

## Discussion

A common theme in cancer metabolism is that cancer-associated enzymes of interest as potential therapeutic targets [36–38] are frequently found to have non-metabolic “moonlighting” functions [39, 40]. In this study, we have demonstrated that MTHFD2 interacts with ribosomal and RNA processing proteins (Fig. 2**,** Table 1), which are also coexpressed with MTHFD2, and give similar transcriptional phenotypes upon shRNA knockdown. These distinct methods provide independent evidence suggesting that MTHFD2 has a previously unrecognized function in RNA metabolism and/or translation, although further experiments are required to confirm these findings, for example using ribosomal profiling, in-depth analysis of alternative splicing, and additional interaction data including reverse Co-IP experiments. The MTHFD2-interacting proteins clearly consist of previously known complexes of proteins with related, but also diverse, functions. We found multiple members of the heterogeneous ribonuclear protein (hnRNP) family of RNA binding proteins, which are of particular interest for several reasons. hnRNPs are known to interact with nascent RNA and control their stability, localization, splicing, and translation, and several family members have been implicated in regulation of cell proliferation and in the endothelial-mesenchymal transition (EMT) process [41, 42], which are also affected by RNAi against MTHFD2 [42–44]. While the consequences of interaction between MTHFD2 and hnRNPs are not yet clear, one possibility is that this interaction may allow nuclear MTHFD2 to exert some control over gene expression; indeed, there are many examples of hnRNP-interacting proteins influencing gene regulation via hnRNPs [45–47]. Moreover, hnRNPs may mediate signals on metabolic status: in particular, hnRNP-E1 has been reported to regulate gene transcription in response to folate starvation [48]. We also observed interactions with components of the small ribosomal subunit, and with several heat-shock proteins (HSPs) that assist with the folding and transportation of target proteins. While HSPs could interact with MTHFD2 simply to assist its own folding, it is also possible that MTHFD2 or other components of the observed complexes bind and that regulate the functions of the HSPs [49]. Interestingly, some of the observed hnRNPs are involved in heat shock responses as well [50], hinting at a common role. We also found interactions with proteins involved in DNA repair and replication (XRCC6 and RPN1), as well as histones (Fig. 2; Table 1, consistent with observations that the protein localize to regions of newly formed DNA [17]. Whether these findings represent more than one distinct function of the MTHFD2 protein, or rather are multiple aspects of a single mechanism, is not yet clear.

Although Co-IP coupled with MS is a powerful methodology for the unbiased detection of protein interactions, it is still a technique with false positives (due to off-target binding of antibodies) and false negatives (due to failure to capture protein-proteins interactions that are of transient nature, or due to other technical reasons). To reduce the impact of false positives, we utilized two antibodies and a CRISPR knockout control. Due to this stringent approach, it is more probable that we have missed some real protein interactors rather than identified false positives. Interestingly, we did not detect interactions between MTHFD2 and other one-carbon metabolism enzymes involved in nuclear dTMP synthesis (SHMT1, SHMT2, MTHFD1, TYMS or DHFR) [51], nor with nuclear lamin to which they are tethered [31]. Such interactions could of course have been missed in our experiments, since we used quite stringent criteria to obtain high-quality interactions and did not perform cross-linking during CoIP. Alternatively, MTHFD2 may reside in a different compartment. From a biochemical perspective, nuclear MTHFD2 would not be expected to contribute to dTMP synthesis, since this process requires CH_2_-THF which MTHFD2 cannot generate on its own, as it lacks the formyl-THF synthase domain present in MTHFD1.

An open question is what drives the recruitment and association of MTHFD2 with cellular RNA-processing protein complexes. Because several of the D2PPI proteins are RNA-binding, one possibility is that MTHFD2 itself is an RNA-binding protein and encounters its partners while bound to specific RNAs. Indeed, a recent large-case study reported that RNA binding proteins tend to CoIP with a large number of other RNA binding proteins [52]. Additionally, MTHFD2 contains Rossman folds, NAD-binding domains that can also bind RNA, as in the example of GAPDH [53]. A number of enzymes have been shown to “moonlight” by binding RNA, including SHMT2 and MTHFD1 [54], and it has been suggested that this may allow metabolic status to influence gene regulation [55]. However, it is also possible that MTHFD2 is not a direct RNA-binding protein, but instead contacts one or more proteins around which the RNA-binding complex is organized. Further work is required to demonstrate whether MTHFD2 binds RNA species directly or indirectly, and understand how the observed protein-protein interactions are formed and function. Further experiments will also be important for validating and extending the findings of this study, such as Co-IP and transcriptomic studies of enzymatically inactive MTHFD2 in diverse nutritional and cell conditions. For example it will be of interest to determine whether the interaction partners of MTHFD2 are modified in response to availability of one-carbon units, growth factors or DNA damage.

## Conclusions

Our study suggests a role for nuclear MTHFD2 in RNA metabolism and translation, besides its established function in mitochondrial folate metabolism. An intriguing possibility is that the MTHFD2-interacting proteins could provide a mechanism whereby MTHFD2 can affect gene expression and cell behaviour, perhaps serving to integrate information on folate metabolism. While the precise mechanism remains to be investigated, such a function would explain the observation that catalytically inactive MTHFD2 protein is sufficient to promote cell proliferation [17]. Future investigation of this non-enzymatic function will be important for pharmaceutically targeting MTHFD2; for example, inhibitors of MTHFD2 dehydrogenase activity may not be effective, and instead drugs that disrupt protein-protein interactions might be required.

## Additional files

Additional file 1: **Table S1.** List of primers used for qRT-PCR in this study. (DOCX 12 kb)

Additional file 2: **Figure S1.** Validation of MTHFD2 knockout cell model. (a) Immunoblot of MTHFD2 in WT and D2-KO clones. Tubulin is shown as loading control. (b) Immunoprecipitation of MTHFD2 using Anti-MTHFD2 antibody from D2-KO and WT cells followed by immunoblotting for MTHFD2. (c) Silver-stained SDS-PAGE of CoIP lysates from WT and D2-KO cells. As indicated the CoIP were performed using either of the anti-MTHFD2 NC or AP antibodies. MTHFD2 bands are indicated by blue squares. As can be seen MTHFD2 were detected in IP samples from WT but not D2-KO cells. (PDF 311 kb)

Additional file 3: Mapped peptides detected by Mass Spectrometry in each of 12 CoIP lysate samples. (XLSX 748 kb)

Additional file 4: Significantly enriched GO Molecular Function and Cellular Component gene sets for 29 identified MTHFD2 protein interactors. (XLSX 15 kb)

Additional file 5: Co-expression scores between MTHFD2 and the D2PPI genes. The gene co-expression were calculated across 8097 human, rat, and mouse datasets using in-house tools, or against ~ 3000 human datasets using SEEK. (XLSX 11 kb)

Additional file 6: List of top data sets exhibiting coexpression between MTHFD2 and the D2PPI and ATF4 gene sets, respectively. (XLSX 17 kb)

Additional file 7: Correlation scores of MTHFD2 against all other genes in Achilles Project. (XLSX 1006 kb)

## Abbreviations

AP: Proteintech 12,270–1-AP anti-MTHFD2 antibody
CoIP: Co-immunoprecipitation
D2-KO: MTHFD2 CRISPR knockout cell line
D2PPI: Set of D2 protein-protein interactors, listed in Table 1.
HNRNP: Heterogeneous ribonucleoprotein
HSP: Heat-shock proteins
IgG: Immunoglubulin G
MS: Mass spectrometry
MTHFD2: Methylenetetrahydrofolate dehydrogenase (NADP+ dependent) 2
NC: Genetex N1C3 anti-MTHFD2 antibody
PPI: Protein-protein interaction
