# Supplementary material for: Protein interaction and functional data indicate MTHFD2 involvement in RNA processing and translation
Source: Cancer Metab. 2018 Sep 27;6:12. doi: 10.1186/s40170-018-0185-4 (PMC6158883; doi:10.1186/s40170-018-0185-4)
Supplement: Supplementary file 1 — Table S1. List of primers used for qRT-PCR in this study. (DOCX 12 kb) [file 40170_2018_185_MOESM1_ESM.docx]

| Gene Name | Forward Primer | Reverse Primer |
| --- | --- | --- |
| MTHFD2 | GATGGCCTCCTTGTTCAGTTG | ATCCTTGTCTGGAGAAACAGCATT |
| SHMT2 | GACACCAGTGTCGCTCTGGATCTG | AGGATAACCCTCCGAGTACTTGTT |
| SHMT1 | AGGAAAGGAGTGAAAAGTGTGGAT | GACACCAGTGTCGCTCTGGATCTG |
| MTHFD1L | CAACATCAAGTGCCGAGCTT | AAGAGGAACACCAGCCGTTA |
| MTHFD1 | GCGCCAGCAGAAATCCTGA | AGGTACTTGCTCCTTCAACTG |
| RPLPO | GGCGACCTGGAAGTCCAACT | CCATCAGCACCACAGCCTTC; |

**Supplementary Table 1**: Primer sequences used for qRT-PCR
