# Supplementary figures and images for: Protein interaction and functional data indicate MTHFD2 involvement in RNA processing and translation
Source: Cancer Metab. 2018 Sep 27;6:12. doi: 10.1186/s40170-018-0185-4 (PMC6158883; doi:10.1186/s40170-018-0185-4)

**A**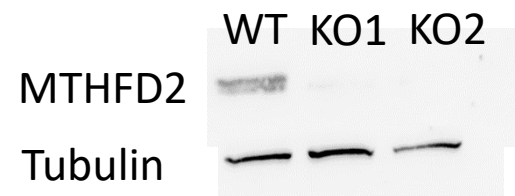**B**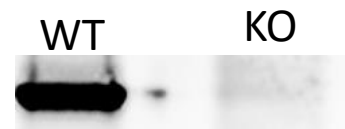**C**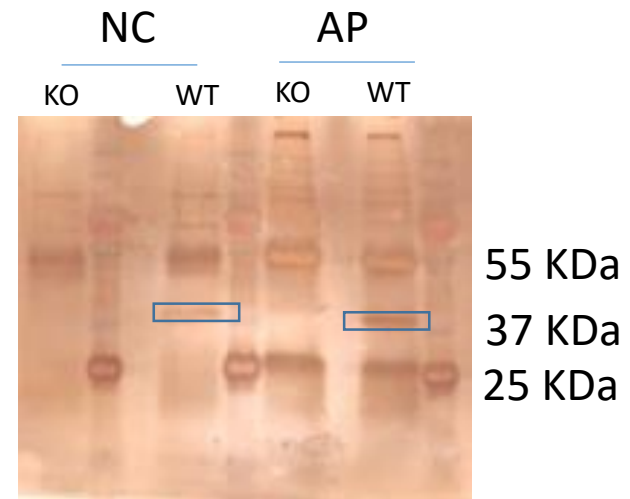

Supplement: Supplementary file 2 — Figure S1. Validation of MTHFD2 knockout cell model. (a) Immunoblot of MTHFD2 in WT and D2-KO clones. Tubulin is shown as loading control. (b) Immunoprecipitation of MTHFD2 using Anti-MTHFD2 antibody from D2-KO and WT cells followed by immunoblotting for MTHFD2. (c) Silver-stained SDS-PAGE of CoIP lysates from WT and D2-KO cells. As indicated the CoIP were performed using either of the anti-MTHFD2 NC or AP antibodies. MTHFD2 bands are indicated by blue squares. As can be seen MTHFD2 were detected in IP samples from WT but not D2-KO cells. (PDF 311 kb) [file 40170_2018_185_MOESM2_ESM.pdf]
